# Optimizing the therapeutic benefits of synriam combined with praziquantel in mice harbouring juvenile and mature *Schistosoma mansoni*

**DOI:** 10.1038/s41598-025-14037-5

**Published:** 2025-08-07

**Authors:** Abdel-Nasser A. Sabra, Maha B. Salem, Samia William, Olfat A. Hammam, Naglaa M. El-Lakkany

**Affiliations:** 1 Pharmacology Department, Theodor Bilharz Research Institute, 1 El-Nile St., Warrak El-Hadar, Imbaba, P.O. box 30, Giza, 12411 Egypt; 2 Parasitology Department, Theodor Bilharz Research Institute, 1 El-Nile St., Warrak El-Hadar, Imbaba, P.O. box 30, Giza, 12411 Egypt; 3 Pathology Department, Theodor Bilharz Research Institute, 1 El-Nile St., Warrak El-Hadar, Imbaba, P.O. box 30, Giza, 12411 Egypt

**Keywords:** Apoptosis, CYP450, Granuloma, Inflammation, Praziquantal, *Schistosoma mansoni*, Synriam, Biochemistry, Biological techniques, Drug discovery, Gastroenterology

## Abstract

Schistosomiasis, a prevalent tropical disease, possess public health challenges, with the standard treatment, praziquantel (PZQ), facing some limitations. Synriam (SYN), an antimalarial medication, has showed promises against schistosomiasis, although in vivo research on its efficacy in preventing infection-related consequences has not been thoroughly explored. This study looked at the effectiveness of SNY-PZQ combination treatment against *Schistosoma mansoni* in mice at various developmental phases, including juvenile (schistosomula) and mature stages. Worm load, egg deposition, parasite maturity, and liver histology were among the key outcomes evaluated. Their modulatory effects on liver injury indicators, proinflammatory cytokines, CYP450 enzymes, and apoptosis in mice infected with mature *S. mansoni* were also investigated. The study was divided into two experimental batches: schistosomula and mature stages, with infected mice from each batch divided into five groups to evaluate SNY, PZQ, and their combination. The SNY-PZQ combination was administered 3 weeks post-infection (PI) for schistosomula-stage infection, and 7 weeks PI for mature-stage infection. When SYN is combined with PZQ in their sub-curative doses (SC), it strengthens the worm killing effects, making it more potent than giving PZQ alone (SC), especially when the dual treatment was given against 7-weeks mature worms (95% vs. 76% for PZQ SC). This was accompanied with almost total eggs elimination and the repair of hepatic granulomatous lesions. Nevertheless, this combination therapy has moderate effectiveness (47% vs. 13% for PZQ SC) when given against 3-weeks juvenile worms. Furthermore, administering this combined therapy to 7-weeks mature worms reduces liver damage as evidenced by decreased oxidative stress, inflammation, and apoptosis, as well as normalization of liver serum enzymes, when compared to PZQ alone, implying that they may contribute to liver fibrosis prevention. Overall, SYN, when combined with PZQ, could improve treatment efficacy, potentially overcoming drug failures, offering a cost-effective strategy for managing schistosomiasis in resource-limited countries.

## Introduction

Schistosomiasis is a neglected tropical disease affecting poor populations in Africa, Asia, the Caribbean, and South America^1^with Sub-Saharan Africa accounting for 90% of the estimated 250 million cases worldwide^2,3^. Schistosomiasis produces enterohepatic morbidity due to severe granulomatous inflammation induced by the host immune system’s reaction to parasite eggs and their released soluble egg antigens (SEA)^4,5^. Granulomas protect host against egg antigen exposure^6^; nonetheless, untreated ones trigger inflammatory responses, leading to scarring, hypertension, bleeding, and hepatic fibrosis^5,7^. Hepatic schistosomiasis can be fatal if not adequately treated, and despite recent research on liver damage healing processes, much remains unclear^8^.

Unfortunately, *Schistosoma mansoni* (*S. mansoni*) infection can disrupt oxidative balance by generating reactive oxygen species (ROS), causing oxidative damage and lipid peroxidation, hindering antioxidant defence and destroying lipids in liver cell membranes^9^. The Nrf2 pathway, which regulates the expression of numerous antioxidant and detoxifying genes, is critical in preventing tissue injury and inflammation^10^. Another important factor is the cytoprotective enzyme; heme oxygenases (HO-1), whose expression is sensitive to pro-oxidant stimuli like heme and nitric oxide^11^.

Hepatic schistosomiasis involves inflammation^12^with the Nuclear factor-kappa B (NF-κB) family playing a crucial role in signalling pathways^13^. NF-κB remains dormant in the cytoplasm when it binds to inhibitory proteins, Iκβs^14^. When activated, NF-κB translocates to the nucleus and regulates inflammatory genes, including tumor necrosis factor (TNF-α) and interleukin-6 (IL-6) in immune cells^15^. During inflammation, cytokines like IL-6 and TNF-α released by macrophages, lymphocytes, and neutrophils can influence the expression of cytochrome P450 enzymes (CYPs), disrupting hepatic drug disposition and potentially altering the pharmacological effects of drugs in inflammatory diseases^16,17^. Additionally, egg deposition in the liver cause DNA damage and liver cell apoptosis^18^. Hepatocyte apoptosis is the initial cellular response to injury, triggering various cellular and cytokine cascades that result in tissue damage, inflammation, fibrosis, and eventually hepatic cirrhosis^19^.

The current treatment for schistosomiasis primarily relies on a single drug, praziquantel (PZQ), which is safe, and effective against adult *Schistosoma* worms of all species^20^. However, the imminent risk of drug resistance and PZQ’s limited efficacy against immature parasites underscore the critical need to prioritize the development of an anti-schistosomiasis drug pipeline^21^. Utilizing drug repurposing is a rapid and cost-effective approach to identify compounds targeting new therapeutic pathways efficiently^22^. People in Africa are commonly co-infected with *Schistosoma spp*. and *Plasmodium spp*. because their regional endemicity overlaps. Notably, clinical trials have explored the potential anti-schistosomal effectiveness of antimalarials like artesunate, artemether, and mefloquine, either alone or in combination, with particular interest in their preclinical activity against young schistosomes^23–25^. Surprisingly, when these synthetic medications are combined with PZQ, this treatment approach encompasses the parasite’s whole life cycle^26,27^. Over time, this approach has been recognized for its ability to demonstrate synergistic effects, ultimately boosting the treatment of helminth infections^28^. Piperaquine phosphate, initially developed in 2011 as an antimalarial drug and marketed as Synriam™ (SYN) by Ranbaxy in Goregaon, India, holds significance in this context^29^. Mossallam et al.^30^ found that SYN significantly reduced *S. mansoni* and improved liver pathology, but its effect on oxidative stress, inflammatory, and apoptotic indicators associated with liver damage has not previously been studied, either alone or in combination with PZQ. In light of this, we conducted the first in vivo assessment of the effects of SNY-PZQ combination therapy against *S. mansoni* at different developmental stages. This covers the schistosomula stage, where PZQ exhibits limited effectiveness, and the mature-stage. The key outcomes assessed included worm load, egg deposition, parasite maturity, and liver histology. Since attaining maturity, *S. mansoni* not only creates ROS, but also affects hepatocellular function, activates inflammatory processes, and induces apoptosis via granuloma formation, with trapped eggs providing antigenic stimulation. Our work is the first to examine how combination therapy affected liver damage indicators, proinflammatory cytokines (NF-kβ, TNF-α, and IL-6), CYP P450 enzymes, and apoptotic markers (caspase-3, Bax, and Bcl-2) in mature *S. mansoni* infection.

## Results

### Combining SYN with PZQ significantly improves parasitological parameters over PZQ alone

#### Effect of SYN-PZQ combination against 3-week-old juvenile worms

Treatment with a full dose of PZQ (1000 mg/kg) showed minimal impact on male and female worm counts in the liver and portomesenteric regions, as well as on the overall worm burden, indicating limited effectiveness against immature worms. In contrast, SYN administered alone at 500 mg/kg significantly reduced male and female worm counts in the liver (*p* = 0.018 and *p* = 0.04, respectively) and in the portomesenteric area (*p* = 0.002 and *p* = 0.045), resulting in a 76.94% decrease in total worm burden compared to the infected untreated group. A lower dose of PZQ (250 mg/kg) failed to produce significant reductions in worm counts in either the liver or portomesenteric region, with only a modest 12.59% decrease in total worm burden. However, the combination of reduced doses of SYN (250 mg/kg) and PZQ (250 mg/kg) significantly lowered male and female worm loads in both the liver and portomesenteric regions by 45.45%, 57.14% and 45.66%, 53.05%, respectively, culminating in a 47.37% reduction in total worm burden. This combination therapy demonstrated significantly greater efficacy than either the full or subcurative dose of PZQ (Table 1).

**Table 1:** The effect of synriam in combination with praziquantel given to mice 3 weeks after *S. mansoni* infection on the total number of males, females, and worms recovered 2 weeks following treatment.

Animal groups	Hepatic		Portomesenteric		Total worms
	Total males	Total females	Total males	Total females	
Infected-untreated	2.75 ± 0.41	1.75 ± 0.37	4.38 ± 0.32	2.13 ± 0.44	28.75 ± 1.41
PZQ (FD)	2.63 ± 0.32 (4.36%)	1.63 ± 0.32 (6.86%)	3.75 ± 0.73 (14.38%)	1.75 ± 0.37 (17.84%)	22.00 ± 1.27 (23.48%) *p* = 0.003^*^
SYN (FD)	1.89 ± 0.24 (31.27%) *p* = 0.018^*^ *p* = 0.049^#^	0.75 ± 0.37 (57.14%) *p* = 0.04^*^	1.13 ± 0.23 (74.20%) *p* < 0.002^*^ *p* = 0.003^#^	0.63 ± 0.32 (70.42%) *p* = 0.045^*^	6.63 ± 0.53 (76.94%) *p* < 0.001^*^ *p* < 0.001^#^
PZQ (SC)	2.50 ± 0.50 (9.09%)	1.50 ± 0.38 (14.28%)	4.00 ± 0.46 (8.67%) *p* = 0.001^$^	1.88 ± 0.30 (11.74%)	25.13 ± 1.22 (12.59%) *p* < 0.001^$^
PZQ (SC) + SYN (SC)	1.50 ± 0.27 (45.45%) *p* = 0.016^*^ *p* = 0.045^#^	0.75 ± 0.37 (57.14%) *p =* 0.04^*^ *p =* 0.045^#^ *p* = 0.049^‡^	2.38 ± 0.42 (45.66%) *p* = 0.033^*^	1.00 ± 0.42 (53.05%)	15.13 ± 1.42 (47.37%) *p* < 0.001^*^ *p* = 0.003^#^ *p* < 0.001^$^ *p* < 0.001^‡^
ANOVA	F = 3.969 *p* = 0.009	F = 1.828 *p =* 0.045	F = 8.477 *p* < 0.001	F = 2.874 *p* = 0.037	F = 52.248 *p* < 0.001

Values are presented as means of 12 mice ± SEM. Values between parentheses represent percent reduction in worms compared with the infected untreated group. Statistical analysis was carried out using one-way ANOVA followed by Tukey *post hoc* test. ^*, #, $, ‡^Significantly different from Infected-untreated, PZQ (FD), SYN (FD) or PZQ (SC) groups at *p* < 0.05, respectively. PZQ (FD): Praziquantel full dose (1000 mg/kg), SYN (FD): Synriam full dose (500 mg/kg), PZQ (SC): Praziquantel subcurative dose (250 mg/kg), SYN (SC): Synriam subcurative dose (250 mg/kg). All mice were killed 5 weeks PI (2 weeks post treatment).

**Fig. 1:**
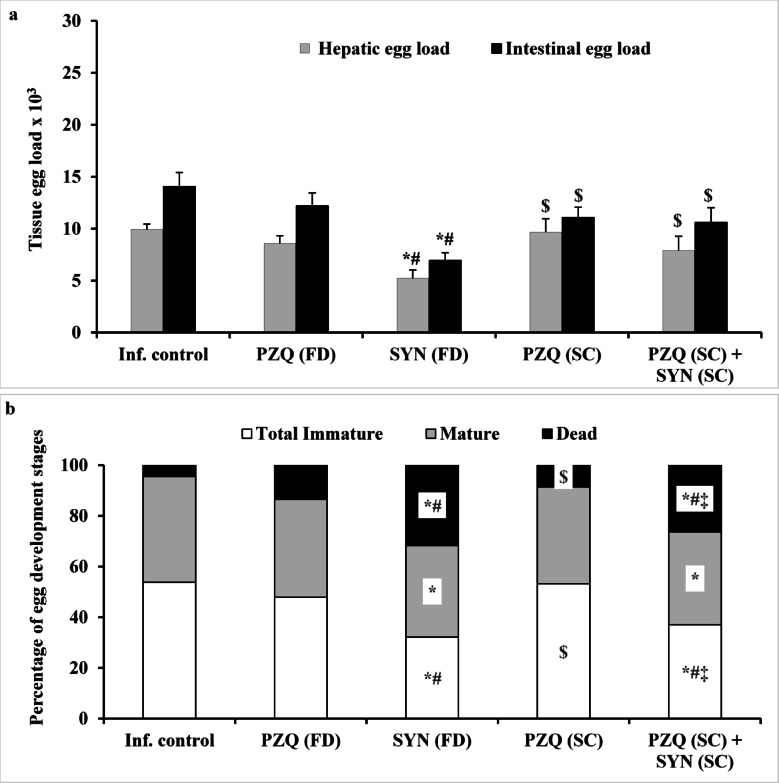
The effect of synriam in combination with praziquantel on tissue egg load (**a**) and percentage of egg development stages (**b**) in mice treated 3 weeks PI (3-week-old juvenile worms). Values are presented as means of 12 mice ± SEM. Statistical analysis was carried out using one-way ANOVA followed by Tukey post hoc test. ^*,#,$,‡^Significantly different from Infected-untreated, PZQ (FD), SYN (FD) or PZQ (SC) groups at *p* < 0.05, respectively. PZQ (FD): Praziquantel full dose (1000 mg/kg), SYN (FD): Synriam full dose (500 mg/kg), PZQ (SC): Praziquantel subcurative dose (250 mg/kg), SYN (SC): Synriam subcurative dose (250 mg/kg). F values were 4.070 and 6.342 for hepatic and intestinal egg loads, respectively, and 15.716, 1.064 and 26.389 for percentage of immature, mature and dead eggs, respectively.

Analysis of tissue egg load and percentage of egg developmental stages (oogram pattern) revealed that treatment with either subcurative or full doses of PZQ alone did not produce a significant difference in overall dead egg count or hepatic and intestinal tissue egg loads compared to the infected untreated group. In contrast, treatment with SYN at 500 mg/kg resulted in a significant reduction in hepatic (47.38%; *p* = 0.009) and intestinal (50.60%; *p* < 0.001) egg burdens, along with a moderate increase in total dead eggs relative to the infected untreated group (31.7% versus 4.5%). Moreover, combining low doses of SYN and PZQ partially enhanced the number of dead eggs (26.38% versus 4.5%); *p* < 0.001), with a modest reduction in hepatic and intestinal egg burdens by 20.44% and 24.58%, respectively, when compared to the infected untreated controls (Fig. 1). These findings show that the limited antischistosomal effectiveness found is mostly due to SYN treatment at the immature (schistosomula) stage.

#### Effect of SYN-PZQ combination against 7-week-old mature worms

Administration of a full dose of PZQ (1000 mg/kg) led to a significant reduction in the total worm burden compared to the infected-untreated group. Specifically, male and female worms in the liver were reduced by 85.28% and 97.09%, respectively, while reductions in the portomesenteric area were 98.72% and 99.10%. This corresponded to a highly significant overall decrease (*p* < 0.001) of 95.99% in total worm burden. Treatment with SYN alone at 500 mg/kg also produced a marked reduction in male and female worm counts in both the liver (*p* < 0.001 and *p* = 0.005, respectively) and portomesenteric regions (*p* < 0.001), leading to a total worm burden reduction of 67.02% compared to untreated controls. When PZQ was given at a lower dose of 250 mg/kg, there was no significant difference in female worm count in the liver, but there was a notable reduction in hepatic male worms (*p* < 0.001), as well as male (85.51%) and female (85.99%) worms in the portomesenteric region (*p* < 0.001). Combining lower doses of SYN (250 mg/kg) and PZQ (250 mg/kg) significantly decreased male and female worm burdens in the liver and portomesenteric areas (86.72%, 97.09% and 96.53%, 100%, respectively), and resulted in an overall worm burden reduction of 95.48%. This combined treatment showed statistically significant improvements (*p* < 0.001) compared to treatment with SYN or PZQ alone **(**Table 2**)**.

**Table 2:** The effect of synriam in combination with praziquantel given to mice 7 weeks after *S. mansoni* infection on the total number of males, females, and worms recovered 2 weeks following treatment.

Animal group	Hepatic		Portomesenteric		Total worms
	Total males	Total females	Total males	Total females	
Infected-untreated	6.25 ± 1.06	2.75 ± 0.48	13.25 ± 0.55	8.92 ± 0.43	31.17 ± 1.17
PZQ (FD)	0.92 ± 0.19 (85.28%) *p* < 0.001^*^	0.08 ± 0.08 (97.09%) *p* < 0.001^*^	0.17 ± 0.57 (98.72%) *p* < 0.001^*^	0.08 ± 0.08 (99.10%) *p* < 0.001^*^	1.25 ± 0.28 (95.99%) *p* < 0.001^*^
SYN (FD)	2.75 ± 0.37 (56.00%) *p* < 0.001^*^	1.08 ± 0.40 (60.73%) *p* = 0.005^*^	3.50 ± 0.57 (73.58%) *p* < 0.001^*^ *p* < 0.001^#^	3.00 ± 0.41 (66.37%) *p* < 0.001^*^ *p* < 0.001^#^	10.*28 ± 1.32* (67.02%) *p* < 0.001^*^ *p* < 0.001^#^
PZQ (SC)	2.58 ± 0.38 (58.72%) *p* < 0.001^*^	1.58 ± 0.34 (42.55%) *p* = 0.014^#^	1.92 ± 0.43 (85.51%) *p* < 0.001^*^ *p* = 0.035^#^	1.25 ± 0.45 (85.99%) *p* < 0.001^*^ *p* < 0.004^$^	7.33 ± 0.27 (76.48%) *p* < 0.001^*^ *p* < 0.001^#^
PZQ (SC) + SYN (SC)	0.83 ± 0.24 (86.72%) *p* < 0.001^*^	0.08 ± 0.08 (97.09%) *p* < 0.001^*^ *p* = 0.014^‡^	0.50 ± 0.19 (96.53%) *p* < 0.001^*^ *p* < 0.001^$^	0.00 ± 0.00 (100%) *p* < 0.001^*^ *p* < 0.001^$^	1.41 ± 0.39 (95.48%) *p* < 0.001^*^ *p* < 0.001^$^ *p* = 0.001^‡^
ANOVA	F = 16.229 *p* < 0.001	F = 12.201 *p* < 0.001	F = 168.125 *p* < 0.001	F = 122.417 *p* < 0.001	F = 194.004 *p* < 0.001

Values are presented as means of 12 mice ± SEM. Values between parentheses represent percent reduction in worms compared with infected untreated group. Statistical analysis was carried out using one-way ANOVA followed by Tukey *post hoc* test. ^*, #, $, ‡^ Significantly different from Infected-untreated, PZQ (FD), SYN (FD) or PZQ (SC) groups at *p* < 0.05, respectively. PZQ (FD): Praziquantel full dose (1000 mg/kg), SYN (FD): Synriam full dose (500 mg/kg), PZQ (SC): Praziquantel subcurative dose (250 mg/kg), SYN (SC): Synriam subcurative dose (250 mg/kg). All mice were killed 9 weeks PI (2 weeks post treatment).

**Fig. 2:**
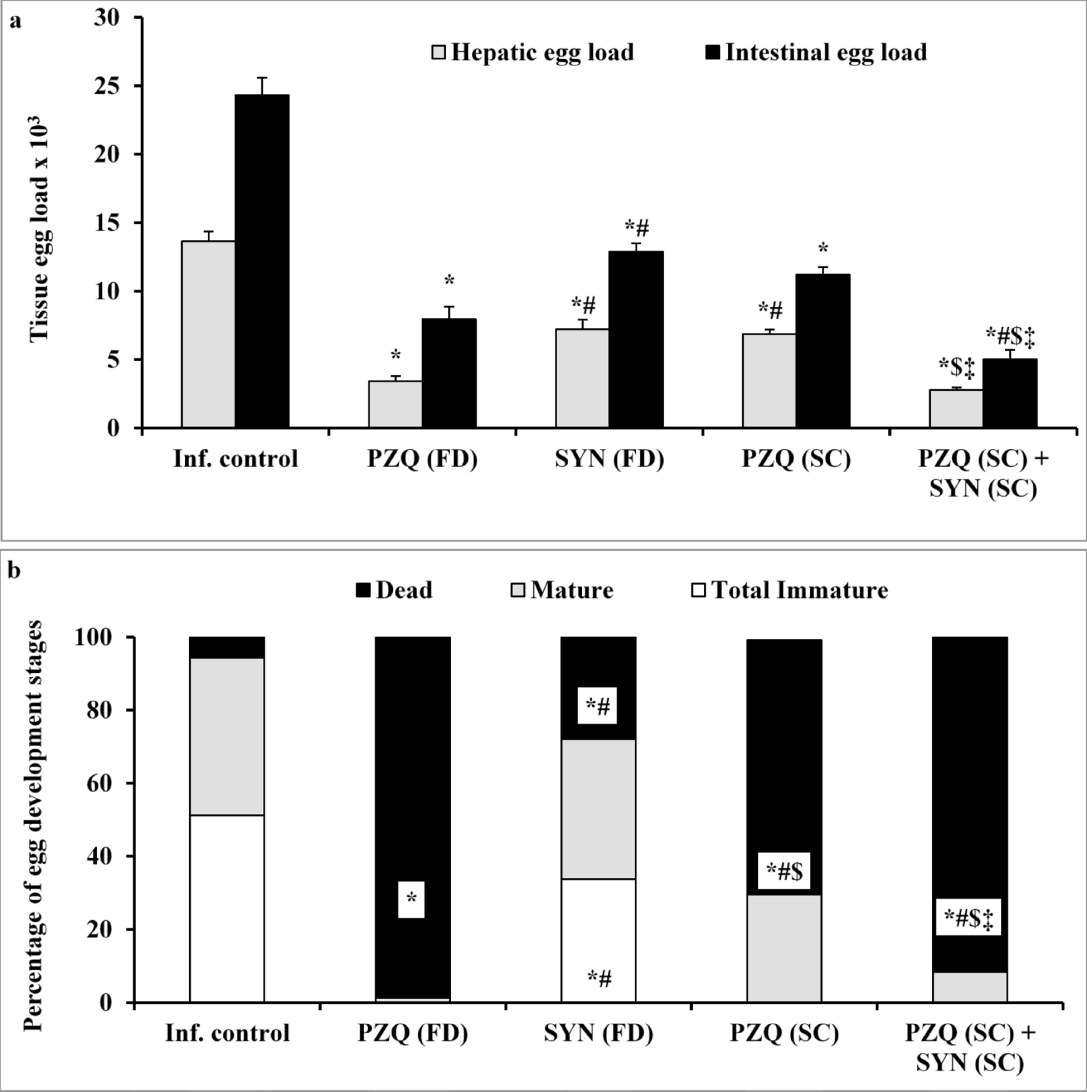
The effect of synriam in combination with praziquantel on tissue egg load (**a**) and percentage of egg development stages (**b**) in mice treated 7 weeks PI (7-week-old mature worms). Values are presented as means of 12 mice ± SEM. Statistical analysis was carried out using one-way ANOVA followed by Tukey *post hoc* test. ^*, #, $, ‡^ Significantly different from Infected-untreated, PZQ (FD), SYN (FD) or PZQ (SC) groups at *p* < 0.05, respectively. PZQ (FD): Praziquantel full dose (1000 mg/kg), SYN (FD): Synriam full dose (500 mg/kg), PZQ (SC): Praziquantel subcurative dose (250 mg/kg), SYN (SC): Synriam subcurative dose (250 mg/kg). F values were 75.465 and 87.747 for hepatic and intestinal egg loads, respectively, and 107.845, 58.305 and 420.231 for percentage of immature, mature and dead eggs, respectively.

Regarding tissue egg load and percentage of egg developmental stages (oogram pattern), administration of a full dose of PZQ (1000 mg/kg) led to a significant reduction (*p* < 0.001) in total immature eggs, along with a 74.95% and 67.27% decrease in hepatic and intestinal egg burdens, respectively. This was accompanied by a total dead egg percentage of 98.75%, compared to the infected untreated group. Treatment with SYN (500 mg/kg) and a lower dose of PZQ (250 mg/kg) also resulted in significant reductions in hepatic and intestinal tissue egg burdens *p* < 0.001 for both), with dead egg rates of 27.92% and 69.58%, respectively. Notably, the combination of SYN (250 mg/kg) and PZQ (250 mg/kg) significantly enhanced the antischistosomal efficacy, as indicated by a marked increase in dead eggs (93.33%), complete elimination of immature eggs, and a pronounced reduction in hepatic and intestinal egg burdens by 79.68% and 79.23%, respectively (*p* < 0.001 for both), when compared to the infected untreated group. This combined treatment also showed a significant improvement over SYN or PZQ monotherapy (Fig. 2).

#### Combining SYN with PZQ greatly alleviates histopathopathogical changes over PZQ alone

**Fig. 3:**
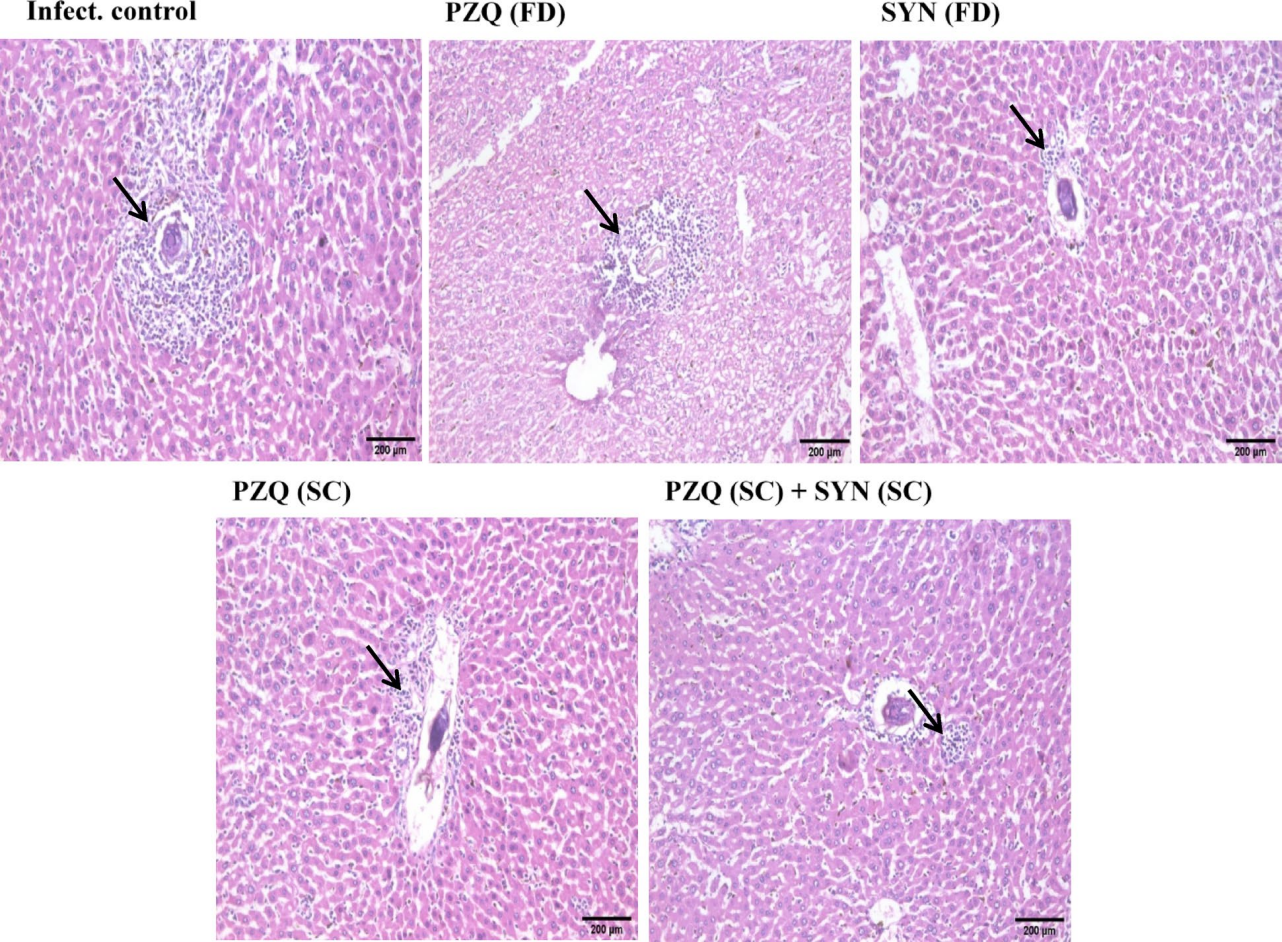
The effect of synriam in combination with praziquantel on histopathological changes in liver sections of mice treated 3 weeks PI (3-week-old juvenile worms), Black arrows indicated inflammation (H & E, ×200). PZQ (FD): Praziquantel full dose (1000 mg/kg), SYN (FD): Synriam full dose (500 mg/kg), PZQ (SC): Praziquantel subcurative dose (250 mg/kg), SYN (SC): Synriam subcurative dose (250 mg/kg).

Figure 3 illustrates the impact of combining SYN with PZQ on liver histopathology during the early stages of *Schistosoma mansoni* infection. Liver tissues from untreated mice infected for 5 weeks showed large, irregularly shaped pre-granulomas containing intact eggs surrounded by chronic inflammatory cells. In contrast, liver sections from mice treated with PZQ at 1000 mg/kg displayed small pre-granulomas with more advanced egg degeneration. Mice administered SYN (500 mg/kg) had smaller pre-granulomas, deteriorated eggs, and less inflammatory cells. PZQ at 250 mg/kg resulted in moderate-sized pre-granulomas with significant inflammatory infiltration. Importantly, the combination of PZQ (250 mg/kg) and SYN (250 mg/kg) resulted in very minor pre-granulomas and a significant reduction in inflammatory cell presence.

**Fig. 4:**
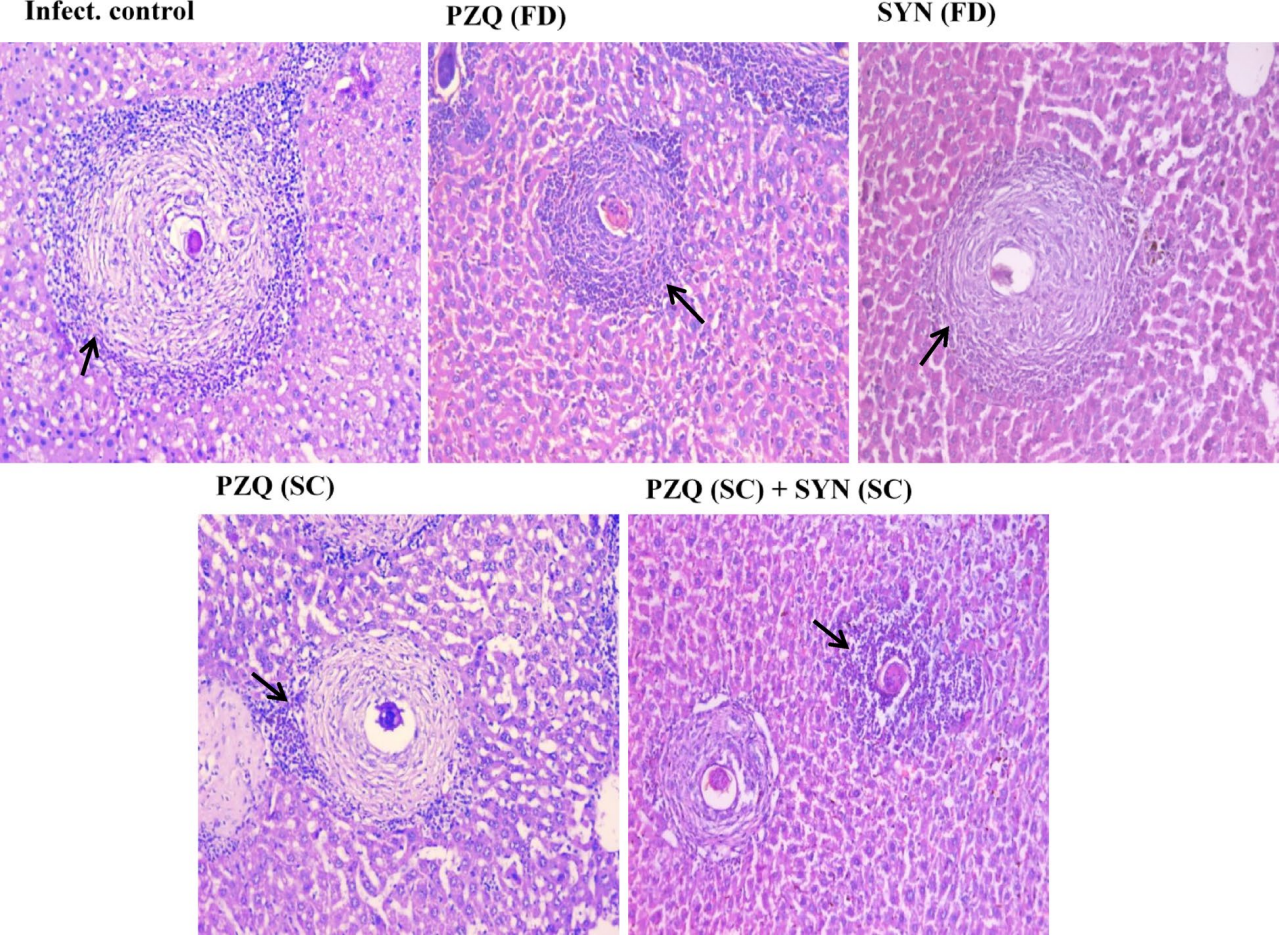
The effect of synriam in combination with praziquantel on histopathological changes in liver sections of mice treated 7 weeks PI (7-week-old mature worms). Black arrows indicated inflammation (H & E, ×200). PZQ (FD): Praziquantel full dose (1000 mg/kg), SYN (FD): Synriam full dose (500 mg/kg), PZQ (SC): Praziquantel subcurative dose (250 mg/kg), SYN (SC): Synriam subcurative dose (250 mg/kg).

In mature stage, liver sections from untreated infected mice showed irregularly shaped large fibrocellular granulomas, averaging 251.13 μm ± 4.31 in diameter. These granulomas were identified by collagenous fibrous tissue surrounding intact ova, with a peripheral zone containing chronic inflammatory cells. Conversely, liver sections from infected mice treated with PZQ at a dosage of 1000 mg/kg displayed smaller, well-defined fibrocellular granulomas (117.78 μm ± 1.57) with increased ova degeneration. Mice treated with SYN (500 mg/kg) exhibited medium-sized circumscribed fibrocellular granulomas (151.64 μm ± 4.93) with more pronounced ova degeneration. PZQ treatment at 250 mg/kg resulted in medium-sized, well-demarcated granulomas measuring 143.51 μm ± 5.15, characterized by increased fibrous tissue and reduced inflammatory cells. Mice treated with SYN (250 mg/kg) and PZQ (250 mg/kg) developed small, well-defined granulomas (124.14 μm ± 3.33) with more fibrous tissue and fewer inflammatory cells (Fig. 4; Table 3).

**Table 3:** Effect of synriam in combination with praziquantel on granuloma diameter and types at mature stage of *S. mansoni* infection.

Animal groups	Granuloma diameter (µm)	Type of granuloma		
		Cellular	Fibrocellular	Fibrous
Infected-untreated	251.13 ± 4.31	38.25 ± 5.16	62.5 ± 4.91	6.25 ± 2.63
PZQ (FD)	117.78 ± 1.57 *p* < 0.001^*^	4.38 ± 1.21 *p* < 0.001^*^	95.63 ± 1.21 *p* < 0.001^*^	0.00 ± 0.000 *p* < 0.001^*^
SYN (FD)	151.64 ± 4.39 *p* < 0.001^*^ *p* < 0.001^#^	0.00 ± 0.00 *p* < 0.001^*^	60.00 ± 2.67 *p* = 0.038^#^	17.50 ± 4.43 *p* < 0.001^*^
PZQ (SC)	143.51 ± 5.15 *p* < 0.001^*^ *p* < 0.001^#^	0.00 ± 0.00 *p* < 0.001^*^	76.25 ± 8.44 *p* < 0.001^#^	8.13 ± 4.00 *p* = 0.001^#^
PZQ (SC) + SYN (SC)	124.14 ± 3.33 *p* < 0.001^*^ *p* < 0.001^$^ *p* = 0.012^‡^	4.00 ± 1.09 *p* < 0.001^*^	96.00 ± 1.09 *p* < 0.001^*^ *p* = 0.033^$^ *p* < 0.001^‡^	0.00 ± 0.00 *p* < 0.001^*^ *p* = 0.001^‡^
ANOVA	F = 187.468 *p* < 0.001	F = 45.407 *p* < 0.001	F = 14.296 *p* = 0.001	F = 9.669 *p* < 0.001

Values are presented as means of 8 mice ± SEM. Statistical analysis was carried out using one-way ANOVA followed by Tukey *post hoc* test. ^*,#,$,‡^Significantly different from Infected-untreated, PZQ (FD), SYN (FD) or PZQ (SC) groups at *p* < 0.05, respectively. PZQ (FD): Praziquantel full dose (1000 mg/kg), SYN (FD): Synriam full dose (500 mg/kg), PZQ (SC): Praziquantel subcurative dose (250 mg/kg), SYN (SC): Synriam subcurative dose (250 mg/kg).

Since schistosome worms reach maturity, *S. mansoni* produces oxidative stress, alters hepatocellular function, stimulates inflammatory processes, and promotes apoptosis via granuloma formation, with trapped eggs providing antigenic stimulation. Accordingly, we selected the second treatment approach (dual treatment against 7-weeks mature worms) to demonstrate for the first time how combination therapy affected liver damage indicators, proinflammatory cytokines (NF-kβ, TNF-α, and IL-6), CYP P450 enzymes, and apoptotic markers (caspase-3, Bax, and Bcl-2).

#### Combining SYN with PZQ markedly mitigates liver injury and restores antioxidant capacity over PZQ alone in mature stage

Infection with *Schistosoma mansoni* in mice led to a significant increase in serum levels of alanine aminotransferase (ALT) and aspartate aminotransferase (AST), by 2.2-fold and 1.48-fold respectively, compared to the normal control group (*p* < 0.001). A notable rise in lipid peroxidation was also observed, with malondialdehyde (MDA) level increasing by 3.53-fold (*p* < 0.001) when compared to normal control group. Additionally, there was a significant reduction (*p* < 0.001) in the hepatic levels of superoxide dismutase (SOD) by 2.12-fold, reduced glutathione (GSH) by 6.6-fold, and Nrf2 and HO-1 by 2.86-fold and 2.56-fold, respectively.

**Fig. 5:**
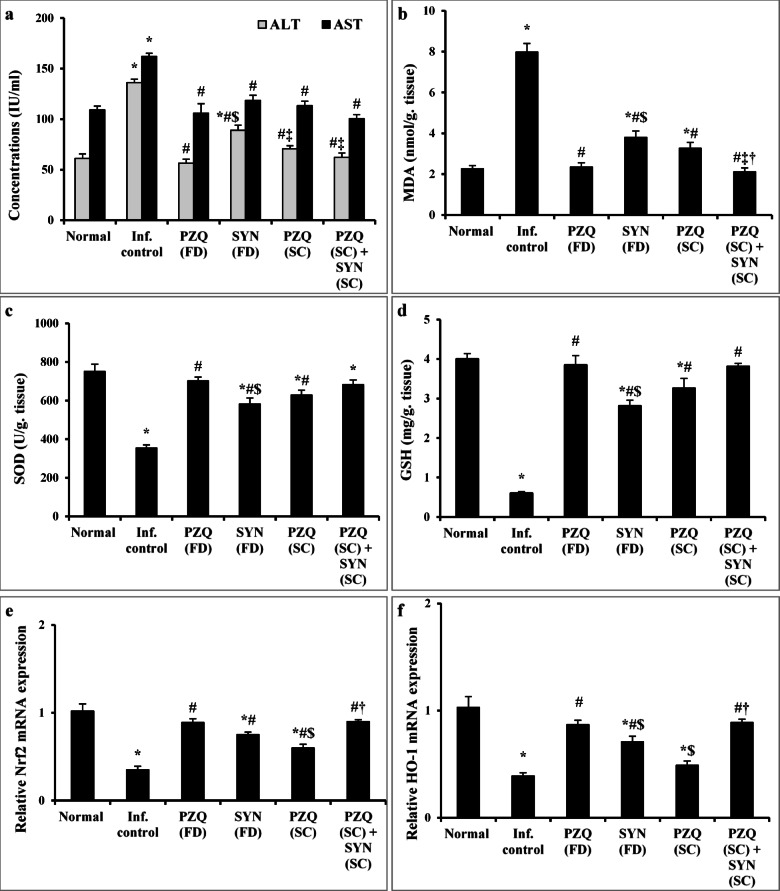
The effect of synriam in combination with praziquantel on liver function markers (**a**), MDA (**b**), SOD (**c**), GSH (**d**), Nrf2 (**e**), and HO-1 (**f**) 9 weeks post infection (2 weeks post treatment). Values are presented as means of 6–12 mice ± SEM. Statistical analysis was carried out using one-way ANOVA followed by Tukey *post hoc* test. ^*,#,$,‡,†^Significantly different from Normal, Infected-untreated, PZQ (FD), SYN (FD) or PZQ (SC) groups at *p* < 0.05, respectively. PZQ (FD): Praziquantel full dose (1000 mg/kg), SYN (FD): Synriam full dose (500 mg/kg), PZQ (SC): Praziquantel subcurative dose (250 mg/kg), SYN (SC): Synriam subcurative dose (250 mg/kg); MDA: Malondialdehyde, SOD: Superoxide dismutase, GSH: Reduced glutathione, Nrf2: Nuclear factor erythroid 2–related factor 2, HO-1: Heme oxygenase-1). F values were 52.624, 17.531, 64.956, 24.747, 58.634, 27.968, and 20.210 for ALT, AST, MDA, SOD, GSH, Nrf2, and HO-1, respectively.

Treatment normalized serum ALT and AST levels in all groups, except in mice receiving SYN at 500 mg/kg, which showed a 12.92% improvement in ALT compared to the untreated infected group (*p* < 0.001). Both SYN (500 mg/kg) and PZQ (250 mg/kg) significantly alleviated oxidative stress caused by the infection, as shown by reduced MDA levels and increased hepatic levels of SOD, GSH, Nrf2, and HO-1. Furthermore, treatment with either a full dose of PZQ (1000 mg/kg) or a combination of SYN (250 mg/kg) and PZQ (250 mg/kg) fully restored antioxidant levels, normalizing MDA, SOD, GSH, Nrf2, and HO-1 (Fig. 5).

#### Combining SYN with PZQ markedly suppresses liver inflammation over PZQ alone in mature stage

**Fig. 6:**
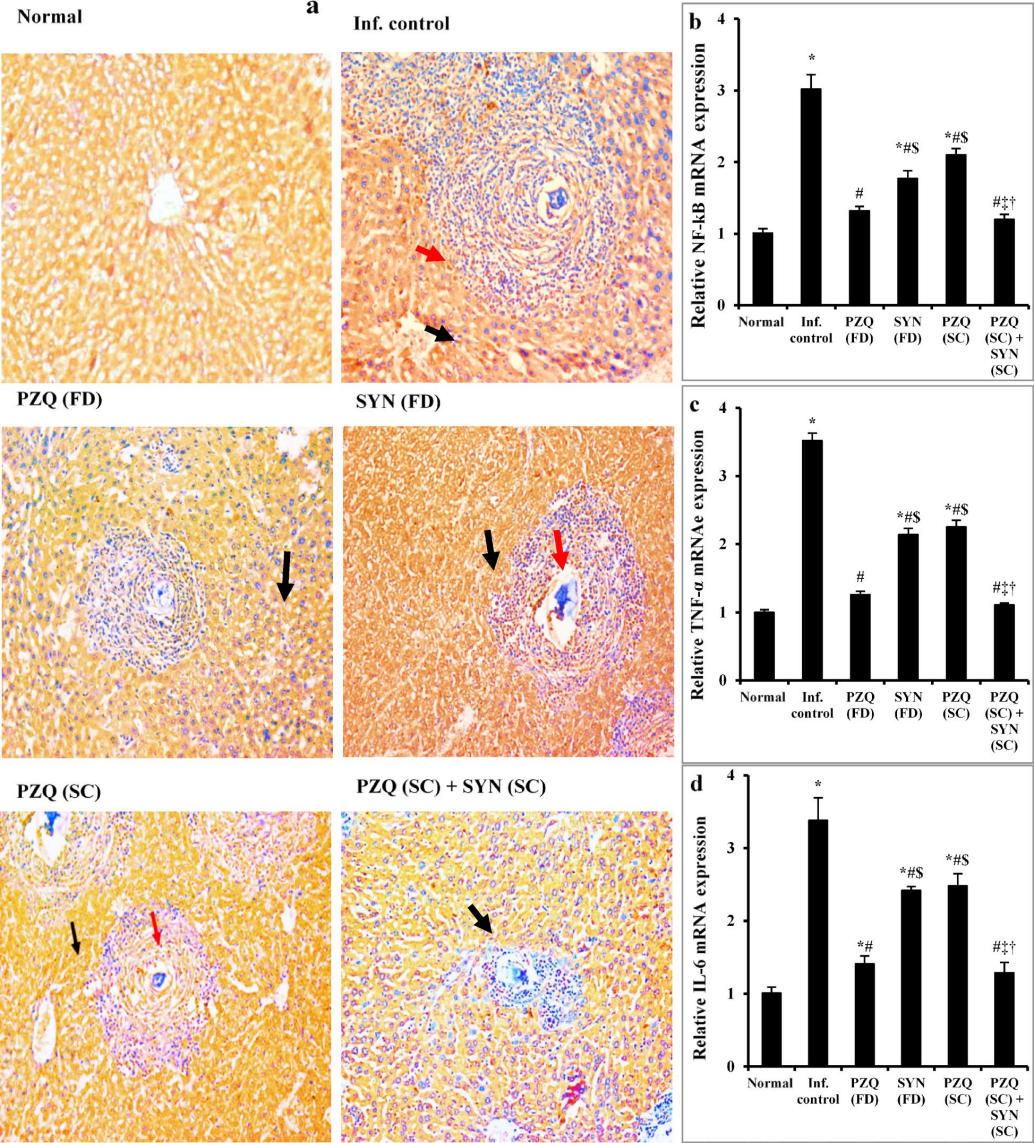
The effect of synriam in combination with praziquantel on immunohistopathological staining for CYP450 (**a**), NF-kβ (**b**), TNF-α (**c**), and IL-6 (**d**) gene expressions in mice liver Sect. 9 weeks post infection (2 weeks post treatment). Black and red arrows indicated CYP450 immunostaining in hepatocytes or in inflammatory cells, respectively (IHC, CYP450, ×200). Values are presented as means of 6 mice ± SEM. Statistical analysis was carried out using one-way ANOVA followed by Tukey *post hoc* test. ^*,#,$,‡,†^Significantly different from Normal, Infected-untreated, PZQ (FD), SYN (FD) or PZQ (SC) groups at *p* < 0.05, respectively. PZQ (FD): Praziquantel full dose (1000 mg/kg), SYN (FD): Synriam full dose (500 mg/kg), PZQ (SC): Praziquantel subcurative dose (250 mg/kg), SYN (SC): Synriam subcurative dose (250 mg/kg), CYP: Cytochrome; NF-kβ: Nuclear factor kappa beta; TNF-α: Tumor necrosis factor alpha; IL-6: Interleukin 6. F values were 48.704, 163.423, and 33.688 for NF-kβ, TNF-α, and IL-6, respectively.

In liver tissue from healthy mice, strong CYP450 expression was observed in hepatocytes, indicated by a brownish cytoplasmic staining, while inflammatory cells showed no detectable expression (Fig. 6a). In contrast, untreated mice infected with *S. mansoni* exhibited moderate CYP450 expression in hepatocytes along with slight expression in inflammatory cells (Fig. 6a). This was accompanied by a significant upregulation (*p* < 0.001) of hepatic NF-κB, TNF-α, and IL-6 gene expression, reflecting increased liver inflammation compared to the normal control group (Fig. 6b–d). Treatment with PZQ at 1000 mg/kg resulted in mild to moderate CYP450 expression in hepatocytes and no expression in inflammatory cells, along with normalization of NF-κB, TNF-α, and IL-6 gene expression (Fig. 6a–d). Similarly, SYN treatment at 500 mg/kg led to moderate CYP450 expression in both hepatocytes and inflammatory cells, accompanied by a significant reduction (*p* < 0.001) in the expression of NF-κB, TNF-α, and IL-6 genes compared to the untreated infected group (Fig. 6b–d). Infected mice receiving a subcurative dose of PZQ (250 mg/kg) displayed moderate CYP450 expression in hepatocytes and mild expression in inflammatory cells (Fig. 6a). This group also showed significant decreases in hepatic NF-κB, TNF-α, and IL-6 gene expression by 30.46%, 36.08%, and 26.63%, respectively, compared to untreated infected controls (Fig. 6b–d). Mice treated with a combination of SYN (250 mg/kg) and PZQ (250 mg/kg) exhibited mild to moderate CYP450 expression in hepatocytes with no expression in inflammatory cells. This combination therapy also restored hepatic NF-κB, TNF-α, and IL-6 gene expression to normal levels (Fig. 6a–d).

#### SYN in combination with PZQ significantly suppresses liver apoptosis over PZQ alone in mature stage

**Fig. 7:**
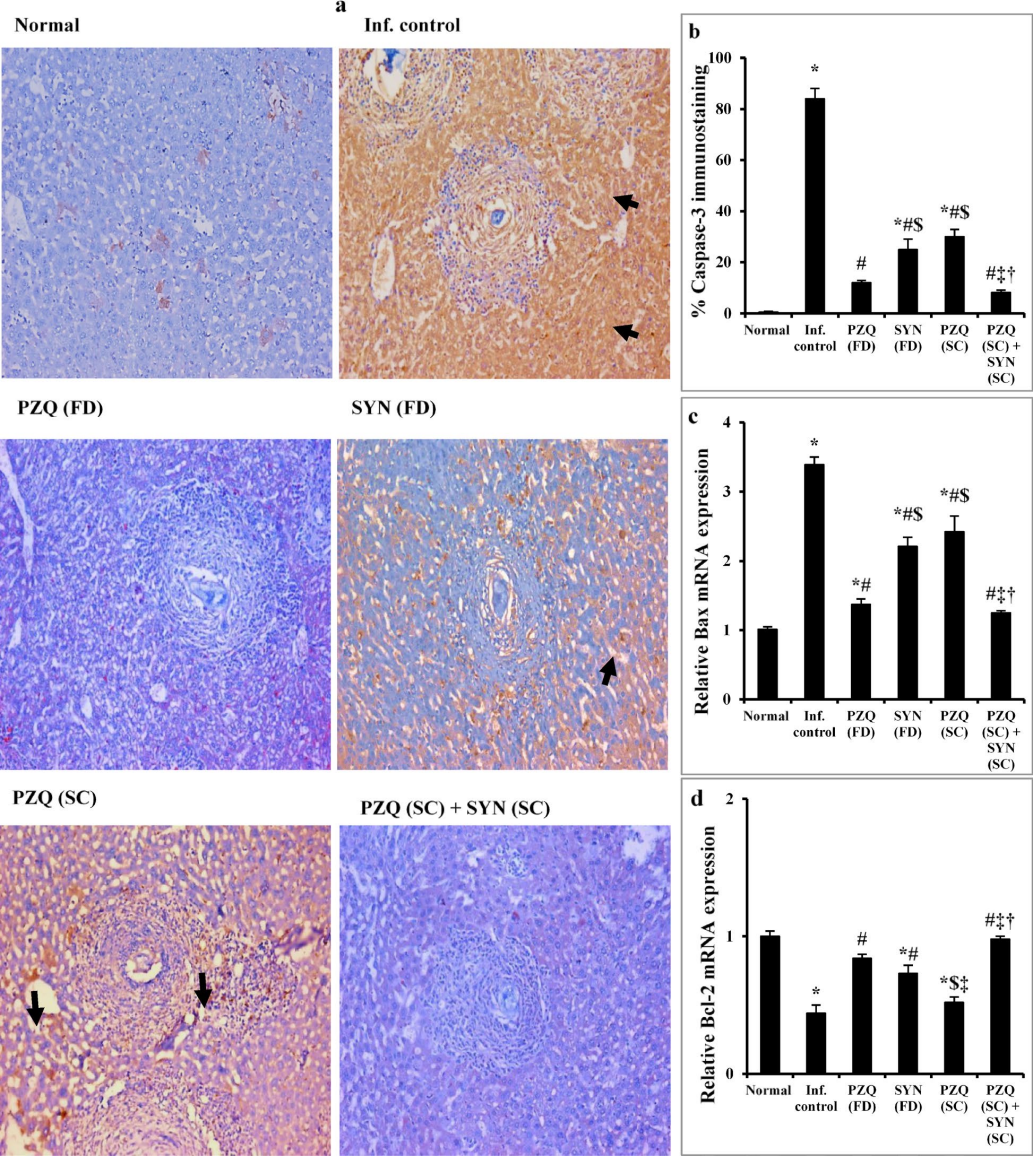
The effect of synriam in combination with praziquantel on immunohistopathological staining for caspase-3 (**a**), % of caspase-3 immunostaining (**b**), Bax (**c**), and Bcl-2 (**d**) gene expressions in mice liver sections, 9 weeks post infection (2 weeks post treatment). Black arrows indicated caspase-3 immunostaining in hepatocytes (IHC, Caspase-3, ×200). Values are presented as means of 6 mice ± SEM. Statistical analysis was carried out using one-way ANOVA followed by Tukey *post hoc* test. ^*, #, $, ‡, †^Significantly different from Normal, Infected-untreated, PZQ (FD), SYN (FD) or PZQ (SC) groups at *p* < 0.05, respectively. PZQ (FD): Praziquantel full dose (1000 mg/kg), SYN (FD): Synriam full dose (500 mg/kg), PZQ (SC): Praziquantel subcurative dose (250 mg/kg), SYN (SC): Synriam subcurative dose (250 mg/kg). F values were 125.582, 54.553, and 29.540 for caspase-3, Bax, and Bcl-2, respectively.

Liver sections from the normal control group showed minimal caspase-3 expression in hepatocytes, indicated by sparse brown cytoplasmic staining (0.5%, Fig. 7a,b). In contrast, liver sections from untreated *S. mansoni*-infected mice exhibited moderate caspase-3 expression (84%) in both hepatocytes and inflammatory cells (Fig. 7a,b). This was associated with a significant upregulation of the pro-apoptotic Bax gene (*p* < 0.001) and a significant downregulation of the anti-apoptotic Bcl-2 gene (*p* < 0.001) compared to the control group (Fig. 7c,d), suggesting enhanced apoptosis in hepatic tissue. Treatment with PZQ at 1000 mg/kg markedly reduced caspase-3 expression to 12% in hepatocytes (an 85.71% reduction from the untreated group), with few brown-stained cells observed (Fig. 7a,b). This was accompanied by normalization of both Bax and Bcl-2 gene expression (Fig. 7c,d). Mice receiving SYN (500 mg/kg) or PZQ (250 mg/kg) showed moderate caspase-3 expression (25% and 30%, respectively) in hepatocytes and inflammatory cells, representing reductions of 70.24% and 64.29% compared to the untreated infected group (Fig. 7a,b). These treatments also significantly decreased Bax expression (*p* < 0.001) and increased Bcl-2 expression (*p* < 0.001 and *p* = 0.01, respectively) (Fig. 7c,d).

Interestingly, combined treatment with SYN (250 mg/kg) and PZQ (250 mg/kg) resulted in low caspase-3 expression (8.17%) confined to hepatocytes, with no expression observed in inflammatory cells (Fig. 7a,b). This represented a 90.27% reduction compared to the untreated group and was significantly lower than in groups treated with SYN or PZQ alone. Furthermore, this combination therapy normalized the hepatic expression levels of both Bax and Bcl-2 (Fig. 7c,d).

## Discussion

Despite its effectiveness against various schistosome species that infect humans, PZQ has limitations and is inefficient in addressing certain associated pathologies^28^. While PZQ is effective against adult schistosomes, it has minimal impact against 2- to 4-week-old juveniles^31,32^and the emergence of PZQ resistance is a growing concern^33^. PZQ’s stage-specificity precludes it from affecting some juvenile parasites, even after repeated treatments. These parasites can grow into adult worms capable of producing eggs, resulting in treatment failure, which is more likely in areas with intense transmission^34^. Moreover, the cost of PZQ is high and not easily affordable by the people who are mostly affected, hindering disease control^35^. Repurposing approved drugs presents a promising avenue for a safe, rapid, and cost-effective alternative in addressing schistosomiasis. This approach holds significant potential, especially considering the slow and uninspiring progress in current drug development efforts for this disease. Drug repositioning expedites clinical trials and regulatory approval, yielding promising drugs for treating schistosomula and adult worms^22^. One instance of successfully repurposed pharmaceuticals is anticancer drugs, notably trametinib and vandetanib, kinase inhibitors, have garnered attention in antischistosomal drug research because of their activities against *S. mansoni*  in vitro and in vivo^36^. Moreover, imatinib’s propensity to disrupt cathepsins, important enzymes involved in protein breakdown and energy metabolism, may increase its antischistosomal potential^37^. Chlorambucil, a nitrogen mustard alkylating agent, has shown promising antischistosomal activity. In vitro, it reduces *S. mansoni* worm viability in a dose-dependent manner. In vivo, it significantly decreases the total worm and egg burdens, demonstrating its highest efficacy against juvenile *S. mansoni*^37^.

Similarly, antimalarial medications are gaining popularity because *Schistosoma spp.* and *Plasmodium spp.* are frequently co-infected in Africa due to their extensive endemicity. Because *Plasmodium* and *Schistosoma* degrade haemoglobin in similar ways, antimalarial medicines are frequently evaluated for antischistosomal activity^38^. While no PZQ-equivalent drug candidate exists, similar drugs can complement each other based on differing mechanisms^39^. Recent studies suggest combinations of agents or antimalarial drugs with PZQ can overcome limitations of PZQ monotherapy^28^. One of these drugs is SYN, also known as Synriam^®^, an antimalarial containing arterolane maleate and piperaquine that was licensed in 2011. Aside from its primary use against malaria, SYN has demonstrated notable anti-schistosomal properties, specifically targeting *S. mansoni*^30,40^implying its potential utility in treating *Schistosoma* and Malaria in co-endemic areas. Nevertheless, there is a shortage of adequate in vivo studies evaluating the efficacy of SYN, whether used alone or in combination with a focus on biochemical and histological changes associated with liver injury, one of the most serious complications of schistosomal infection.

Based on the foregoing, this study investigates the efficiency of SYN in combination with PZQ against juvenile (schistosomula), and mature stages by assessing worm load, egg deposition, parasite maturity, and liver histology. When SYN is combined with PZQ in subcurative doses (SC), the worm killing effects are strengthened, making it more potent than giving PZQ SC alone, especially when the dual treatment was given against 7-week-old mature worms (95% worm reduction vs. 76% for PZQ SC). Furthermore, the combined treatment regimen had a significant impact on *S. mansoni* eggs, resulting in nearly complete egg removal from tissues and minimal histological changes in the liver. This could be attributed to the fact that 97%−100% of female worms were eradicated, and the few remaining worms retrieved from groups receiving the SYN-PZQ combination were nearly sterile and incapable of oviposition. Female schistosomes are more vulnerable to antimalarial medicines than male schistosomes^41,42^. These promising findings are most likely due to the combined actions of antimalarial SYN on the schistosomula and adult stages, as well as schistosomicidal PZQ on the adult stages.

On the other hand, when SYN-PZQ combination is administered to 3-week-old juvenile worms, it is fairly effective (47% worm reduction vs. 13% for PZQ SC), with a slight increase in the number of dead eggs (26.38% vs. 4.5%), as well as a slight reduction in hepatic and intestinal egg burdens of 20.44% and 24.58%, respectively, when compared to infected untreated controls. These data indicate that the moderate antischistosomal efficacy shown is mostly attributable to SYN effect against the schistosomula stage. Preclinical studies show that antimalarial drugs are especially effective against juvenile schistosomes^24,38,43^indicating that concomitant therapy with PZQ may be complimentary. Experimental and clinical trials have approved the antischistosomal potency of antimalarials such as artesunate, artemether, mefloquine, and dihydroartemisinin-piperaquine for the treatment of schistosomiasis, either alone or in combination with PZQ^23,26,44–47^.

Furthermore, the effect of the SYN-PZQ combination in mitigating infection-associated complications caused by schistosome maturation is being investigated by looking at biochemical abnormalities linked with liver damage, as well as proinflammatory cytokines, CYP450 enzymes, and apoptotic markers. In the current study, mice infected with *S. mansoni* developed numerous hepatic granulomas around the accumulated schistosome eggs, affecting hepatocellular function and causing widespread distortion of hepatocellular cords and the lobular structure. Herein, these histopathological changes are associated with impairment of hepatocyte integrity, as evidenced by an increase in serum liver enzymes ALT and AST, as well as oxidative injury, as evidenced by decreased GSH and SOD levels and increased MDA, which is consistent with the findings of Sabra et al.^48^. Treating *S. mansoni*-infected mice with SYN (500 mg/kg) or PZQ (250 mg/kg) alone led to substantial moderate reductions in the total number of worms and female worms, along with decreases in the percentage of immature and mature eggs, hepatic and intestinal tissue egg loads. There was also a notable increase in the percentage of dead eggs, which was associated with moderate healing in hepatic granulomatous lesions. Furthermore, both SYN (500 mg/kg) and PZQ (250 mg/kg) treatments dramatically improved several biochemical markers, including reducing elevated ALT, AST, and MDA levels and replenishing depleted GSH, SOD, HO-1, and Nrf2. Nonetheless, PZQ outperformed SYN in terms of therapeutic effectiveness across all parameters tested. Interestingly, when PZQ was combined with SYN at subcurative doses of 250 mg/kg each, the effect was greater than that of the reduced PZQ dose alone, suggesting a synergistic effect of the two drugs. This combined therapy greatly reduced worm numbers, eliminated females and immature eggs, decreased egg burdens, and healed hepatic granulomatous lesions, resulting in more degenerated eggs, less inflammatory cells, and normal liver enzymes and oxidative stress markers. The study reveals that PZQ and SYN maintain liver cell integrity by preventing toxic worm metabolites, interrupting egg deposition, and directly affecting mature miracidia within egg granulomas, thus preventing SEA emission. Furthermore, removing females may heal hepatic granulomatous lesions by preventing oviposition^26^. The common mechanism of action responsible for the effects of PZQ and SYN on both schistosomes and malaria parasites remains unknown. PZQ is known to induce severe spasms and paralysis of worm muscles, which is likely accompanied by a rapid influx of Ca^2+^ into the schistosome^49^. Meanwhile, it is thought that SYN, like other antimalarial drugs, may interact with haemin (a by-product of hemoglobin degradation) and reduce endoperoxide bridge, producing carbon-centered free radicals that alkylate parasite proteins, causing cell death^50^. Schistosomes, like malaria parasites, require host hemoglobin to survive and have cathepsins that degrade it, resulting in pigment production in their intestines^51^. SYN, as in malaria parasites, may be activated within schistosomes by heme or iron compounds, resulting in hazardous chemicals and free iron radicals, a process that merits further exploration.

Infection with *S. mansoni* not only produces ROS, but it also triggers inflammatory processes through granulomas formation, with trapped eggs providing antigenic stimulation. Periovular granulomas develop by successively attracting inflammatory and immune cells to infection sites^52^. The NF-κB family plays a crucial role in the inflammatory response^13^. When activated, NF-κB regulates inflammatory genes, including TNF-α and IL-6, in various immune cells. Cross-talk between cytokines and chemokines activates hepatic stellate cells, leading to increased inflammation, granuloma formation and regulation of schistosoma-induced immunopathology^15,53^. Herein, both SYN (500 mg/kg) and PZQ (250 mg/kg) exhibited significant anti-inflammatory effects, reducing NF-кB and inflammatory cytokines; TNF-α and IL-6. This anti-inflammatory response was also supported by a decrease in CYP450 immunostaining, since inflammation regulates CYP450 activity via a number of intricate transcriptional and post-transcriptional processes^54^. In this study, treatment with PZQ full dose or a combination of SYN and PZQ normalized CYP450 immunostaining and inflammatory markers, suggesting the removal of trapped eggs from the liver, prevention of SEA release, and suppression of the inflammatory response. Another possible explanation is that drugs have potential anti-inflammatory characteristics; this is an area that warrants additional exploration. Inhibiting inflammatory mediator release is crucial for reducing fibrogenesis in schistosomiasis-related liver pathology^55^. Based on this, the combination of SYN and PZQ may help prevent the progression of liver injury to fibrosis.

Inflammation leads to hepatocyte apoptosis, a common cell death regulated by the caspase cascade^56^which inhibits the production and secretion of inflammatory cytokines by inactivating specific kinases and transcription factors^57^. Cell apoptosis involves key players like Bcl-2, caspase-3, and Bax. Caspase-3 is crucial for apoptosis, regulated by Bcl-2 proteins^58^. Bax, a pro-apoptotic protein, facilitates cytochrome c release through the mitochondrial pathway, thereby activating caspase-3. According to Song et al.^59^schistosome infection increases apoptosis marker proteins in liver tissues, which correlate with liver injury/fibrosis pathology. This implies that cell death contributes to the progression of liver fibrosis. In our study, untreated mice infected with *S. mansoni* demonstrated apoptosis, indicated by elevated caspase-3 immunostaining and hepatic Bax gene expression, along with reduced Bcl-2 gene expression. Administering SYN (500 mg/kg) or PZQ (250 mg/kg) alone significantly reduced liver apoptosis; however, the full dosage of PZQ or a combination of SYN (250 mg/kg) and PZQ (250 mg/kg) had the greatest effect on liver apoptosis reduction. Furthermore, research indicates that SYN (250 mg/kg) and PZQ (250 mg/kg) have a greater effect in reducing liver damage, which is at least mostly due to their capacity to decrease hepatic inflammation and apoptosis.

This is the first study to examine the combined effect of SYN and PZQ on distinct developmental stages of *S. mansoni* infection, such as 3-week-old juvenile worms and 7-week-old mature worms. SYN alone has modest antischistosomal activity; nevertheless, when combined with PZQ in reduced doses, it boosts the antischistosomal impact, which is even higher than giving PZQ in its reduced dose alone, particularly when the dual treatment was administered at the mature stage of infection, suggesting that the two drugs may work synergistically. The combined administration of PZQ and SYN in reduced dosages improved therapeutic effectiveness over either drug alone; this is supported by the nearly complete eradication of adult worms, and eggs, along with the healing of hepatic granulomatous lesions, as confirmed histologically. Importantly, this combination therapy has the greatest impact on lowering female worms, which may disrupt egg deposition and the completion of the schistosome life cycle. Furthermore, this study is the first to show that the combination treatment administered at the mature stage of infection reduces liver damage as evidenced by decreased oxidative stress, inflammation, and apoptosis, as well as normalization of liver serum enzymes when compared to PZQ alone, implying that they may contribute to preventing the development of liver fibrosis. This might be owing to the medications’ multiple actions in targeting schistosome worms, eggs, and toxins. Another probable explanation is that both medications may have anti-inflammatory characteristics; this is an area that might be further investigated. One limitation of this study is the lack of exploration into the therapeutic advantages of this combination therapy in settings where both juvenile and adult parasites coexist through continued infection, which will be addressed in our future research. Overall, SYN could possibly be employed as an adjuvant therapy in conjunction with PZQ at lower doses in the treatment of human schistosomiasis, avoiding potential treatment failures in high transmission areas while being cost-effective in poor countries.

## Materials and methods

### Animals and infection

Adult male Swiss albino mice (20–22 g) acquired from the Schistosome Biology Supply Center of TBRI, Egypt, were kept in controlled settings, fed a regular diet, and given free access to water. *S. mansoni* (Egyptian strain) cercariae infection was performed using the body immersion technique with 80 ± 10 cercariae/mouse collected from infected snails at SBSC^60^.

### Drugs and chemicals

SYN (Synriam^™^, Lot. NO. 2841466) was purchased from Ranbaxy Laboratory Limited in India, and PZQ (Distocide^(R)^; EIPICO, Egypt) was utilized. Both drugs were administered as fresh suspensions in distilled water with 2% Cremophore-El (Sigma-Aldrich, USA) by oral gavages through a stainless-steel cannula.

### Experimental groups

The experimental design was divided into two batches, each corresponding to a distinct developmental stage of *S. mansoni*: schistosomula-stage infection, in which all treatments target juvenile worms 3 weeks PI, and mature-stage infection, in which all treatments target mature worms 7 weeks PI. In each batch, mice infected with *S. mansoni* were divided into five groups of twelve mice each: (I) infected untreated (receiving only the drug vehicle), (II, III) treated with PZQ and SYN at doses of 1000 mg/kg and 500 mg/kg, respectively, administered over two consecutive days, (IV) treated with a single dose of 250 mg/kg PZQ, and (V) treated with both SYN and PZQ at a single dose of 250 mg/kg each. The SNY-PZQ combination therapy was given at stage-specific intervals: 3 weeks post-infection (PI) for immature infections and 7 weeks PI for mature infections. Worm load, egg deposition, parasite maturity, and liver histology were the main parameters evaluated for the efficacy of combination SYN-PZQ therapy against immature and mature *S. mansoni*.

Our study also looked at how combination treatment altered liver damage indicators, proinflammatory cytokines (NF-kβ, TNF-α, and IL-6), CYP P450 enzymes, and apoptotic markers (caspase-3, Bax, and Bcl-2) in mature *S. mansoni* infection. As a result, a group of uninfected and untreated mice was included for testing liver injury and oxidative stress markers, as well as for immunohistochemistry. Mice were euthanised by quick decapitation. Blood samples (~ 0.50–0.75 ml/mouse) were collected, and sera (~ 0.3–0.5 ml/mouse) were separated by centrifugation at 1850 ×g for 10 min and kept at -80 °C for analysis of liver damage biomarkers. In addition, the livers were removed, promptly placed on ice, and separated into three portions. The first portion (~ 0.2–0.5 g/mouse) was used for parasitological examination, the second (one g) was fixed in formalin for histological and immunohistochemical studies, and the third (one g) was homogenized with a Pro Scientific Inc. homogenizer in 100 mM potassium phosphate buffer (1:4 w/v; pH 7.4) and centrifuged at 10,000 ×g for 1 h at 4 °C. The supernatants were harvested and chilled in Eppendorf vials at -80 °C for oxidative stress markers detection and RNA extraction.

### Parameters of assessments

#### Parasitological parameters

Following euthanasia, mice were subjected to hepatic and portomesenteric vessel perfusion for worm recovery and subsequent quantification^61^. After perfusion, a small portion (about 0.2 g) of liver or intestinal tissues was taken from each mouse and incubated with 5 ml of 5% potassium hydroxide (KOH) solution at 37 °C for 24 h, until the tissue was completely digested. For each mouse, two 100 µl samples were examined using a Zeiss binocular microscope (Carl Zeiss Microscopy GmbH 07745 Jena, Germany). The total egg loads in hepatic and intestinal tissues were determined by multiplying the average number of eggs counted in each 100 µl sample by the total KOH volume and then dividing by the sample weight in grams, providing the number of eggs per gram of tissue^62^. The percentage reduction in worm/egg burden was calculated using the formula: % reduction = [(Number of worms/eggs in the control group) - (Number of worms/eggs in the treated group)] / (Number of worms/eggs in the control group) × 100. Additionally, the percentage of egg developmental stages (oogram pattern) in small intestinal tissues was examined in accordance with Pellegrino et al.^63^. In brief, five centimetres of the middle section of the small intestine were longitudinally opened with fine scissors and thoroughly cleaned with saline to eliminate debris. Three 1-cm tissue fragments were then sliced, carefully wiped dry on filter paper, and inspected using a Zeiss binocular dissecting microscope. Each tissue fragment was examined for the presence of *S. mansoni* eggs, and eggs were categorized into three developmental stages based on morphological criteria observed under light microscopy. (1) Immature eggs, classified as stages I-IV (based on the size of their embryos), have a thin shell and an incompletely formed embryo. The contents seem granular or amorphous, with no visible miracidium. (2) Mature eggs include a fully developed ciliated miracidium. These eggs have a well-defined form and visible internal motion in fresh preparations, indicating viability. (3) Dead eggs appear collapsed, dark brown to black, or semi-transparent, with no obvious internal structures. These eggs often contain granular contents and lack the characteristics of viable embryos. For each tissue piece, the number of eggs in each category was counted, and the percentage of each developmental stage was calculated. The mean percentages across the three fragments were used for statistical analysis.

#### Liver histology and granuloma measurement

The livers of mice were fixed in buffered formalin, and paraffin blocks were prepared. Section (5 μm thick) were cut and each mouse had five liver slices stained with hematoxylin and eosin (H&E; magnification ×200). The sizes of hepatic granulomas (30 per mouse) were measured using a computerized image analysis system (Axiovision version 4.8, Zeiss Germany). An ocular micrometer was employed to quantify non-contiguous granulomas, each containing a single egg (with intact or degenerated miracidia). The ocular micrometer is a standard tool for measuring granulomas, allowing for precise measurement under a microscope. The mean diameter of each granuloma was estimated by taking two measurements of the lesion at right angles^64^. Egg viability and granuloma cell composition were both microscopically investigated in the same liver slices.

#### Biochemical parameters

Serum ALT and AST, which serve as indicators of liver damage, as well as oxidative stress markers including hepatic GSH, SOD, and MDA were analyzed spectrophotometrically using an enzymatic colorimetric kit (Biodiagnostics, Egypt), according to the manufacturer’s instructions.

#### Quantitative RT-PCR analysis

Total RNA extraction was carried out from liver tissues in a RNase-free environment using the RNeasy Mini kit (Qiagen). Real-time PCR reactions were conducted using the StepOne™ Real-Time PCR System and 10 µl of 2× PowerUp™ SYBR™ Green/ROX PCR Master Mix (Applied Biosystems, ThermoFisher Scientific, USA) according to the manufacturer’s protocol to assess the expression of NF-kβ, IL-6, TNF-α, Nrf2, HO-1, Bax, and Bcl-2. The primer sequences are provided in Table 4. Relative expression levels of NF-kβ, IL-6, TNF-α, Nrf2, HO-1, Bax, and Bcl-2 were calculated using the comparative cycle threshold (Ct) method (2^−ΔΔCT^), normalized to β-actin as a control^65^.

**Table 4:** Primer sequences for quantitative real-time PCR analysis.

Target gene(s)	Amplicon length (bp)	Accession number	Primer sequence
NF-kβ	78	XM_006526743.5	Forward primer: 5′-CTGGTGGACACATACAGGAAGAC-3′
			Reverse primer: 5′-ATAGGCACTGTCTTCTTTCACCTC-3′
IL-6	542	NM_001314054.1	Forward primer: 5′ CTGGTGACAACCACGGCCTTCCCTA-3′
			Reverse primer: 5′- ATGCTTAGGCATAACGCACTAGGTT-3′
TNT-α	246	NM_001278601.1	Forward primer: 5′-ACCCTCACACTCACAAACCA-3′
			Reverse primer: 5′-GGCAGAGAGGAGGTTGACTT-3′
Nrf2	225	XM_007965441.2	Forward primer: 5’-ATGATGGACTTGGAGCTGCC-3′
			Reverse primer: 5′-TTGTAACTGAGCGAAAAAGGCTTT-3′
HO-1	127	XM_003821507.3	Forward primer: 5′-TTCAGAAGGGCCAGGTGACC-3′
			Reverse primer: 5′-AAGTAGACAGGGGCGAAGACTGG-3’
Bax	109	NM_007527.4	Forward primer: 5′-CCCGAGAGGTCTTTTTCC-3′
			Reverse primer: 5′-GCCTTGAGCACCAGTTTG-3′
Bcl-2	84	NM_009741.5	Forward primer: 5′-CCTGGCTGTCTCTGAAGACC-3′
			Reverse primer: 5′-CTCACTTGTGGCCCAGGTAT-3′
Beta actin	118	NM_007393.5	Forward primer: 5′-GGGAATGGGTCAGAAGGACT-3′
			Reverse primer: 5′-CTTCTCCATGTCGTCCCAGT-3′

#### Immunohistochemical examinations (IHC) for CYP450 and caspase-3

Immunohistochemical analysis was performed on 5 μm thick paraffin-embedded tissue slices to examine CYP450 and caspase-3 expression levels. To expose the antigens, slices were pre-treated with proteinase K (Agilent Dako, USA), washed in phosphate-buffered saline for 5 min, and then incubated with primary antibodies against CYP450 and caspase-3 (Santa Cruz Biotechnology, USA) for 60 min at 37 °C. After a PBS wash, a secondary antibody (Agilent Dako, USA) was applied for an additional 60 min. The reaction was visualized using 3,3’-diaminobenzidine (DAB) chromagen (Agilent Dako, USA). The percentages of CYP450 and caspase-3 positive cytoplasmic brown stained cells in each mouse were semi-quantitatively evaluated in ten consecutive fields (magnification ×200) using Zeiss light microscopy (Carl Zeiss Microscopy GmbH 07745 Jena, Germany).

### Statistical analysis

The data are presented as Mean ± SEM. Statistical analysis was carried out using a one-way ANOVA test followed by Tukey’s *post-hoc* test to evaluate the significance differences among the mean values of various groups. The analysis was conducted using the SPSS software, version 16.0 (Chicago, IL, USA). Results were considered statistically significant at a *p*-value less than 0.05.

## Data Availability

The datasets used and/or analyzed during the current study available from the corresponding author on reasonable request.
